# Seasonal Variation, Circadian Patterns, and Night Eating Syndrome Among Older Adults: The Mediating Role of Sleep Quality

**DOI:** 10.3390/jcm15208163

**Published:** 2026-10-20

**Authors:** Noha Gamal El-Sayed Ghoniem, Mona Metwally El-Sayed, Basema Alharbi, Wagida Wafik, Amira Mohammed Ali, Sahar Mohammed Mohammed Aly, Fatma Mohammed Ahmed

**Affiliations:** 1 Department of Gerontological Nursing, Faculty of Nursing, Zagazig University, Zagazig 44519, Egypt; naha.gamal@zu.edu.eg; 2 Department of Community, Psychiatric, and Mental Health Nursing, College of Nursing, Qassim University, Buraydah 52571, Saudi Arabia; m.mona@qu.edu.sa; 3 Department of Community Health Nursing, College of Nursing, Zagazig University, Zagazig 44519, Egypt; wkamel@kku.edu.sa (W.W.); fmabdelfatah@nursing.zu.edu.eg (F.M.A.); 4 Department of Psychiatric Nursing and Mental Health, Faculty of Nursing, Alexandria University, Smouha, Alexandria 21527, Egypt; amiramali@alexu.edu.eg; 5 Nursing Leadership and Management, Makkah National College, Makkah 21995, Saudi Arabia; s.aly@mnc.edu.sa; 6 Nursing Leadership and Management, Faculty of Nursing, Port-Said University, Port-Said 42526, Egypt

**Keywords:** seasonal variation, circadian pattern, night eating syndrome, sleep quality, older adults, mediation analysis, chronotype, cross-sectional study

## Abstract

**Objective**: We aimed to examine whether sleep quality mediates the relationship between seasonal variation, circadian pattern, and night eating syndrome in community-dwelling older adults. **Methods**: A cross-sectional study was conducted with 336 community-dwelling older adults (aged ≥ 60 years) recruited from geriatric social clubs. Participants completed validated instruments, including the Global Seasonality Scale (GSS), Morningness–Eveningness Questionnaire (MEQ), Night Eating Diagnostic Questionnaire (NEDQ), and Pittsburgh Sleep Quality Index (PSQI). Path analysis with bootstrapping (5000 resamples) was used to estimate direct and indirect associations among the study variables. **Results**: The mean age was 66.8 ± 5.9 years (56.8% male). Moderate to severe seasonal variation was observed in 68.2% of participants, while 85.1% reported poor sleep quality. Morning circadian pattern predominated (56.9%), and 23.2% met criteria for full night eating syndrome. Path analysis identified a significant indirect association between seasonal variation and circadian pattern through sleep quality (B = −0.038, 95% CI [−0.051, −0.026]), corresponding to 41% of the total modeled association. Similarly, a significant indirect association between seasonal variation and night eating syndrome through sleep quality was observed (B = 0.053, 95% CI [0.037, 0.068]), corresponding to 37% of the total modeled association. All direct and indirect paths were statistically significant (*p* < 0.001). **Conclusions**: Sleep quality was significantly involved in the observed relationships between seasonal variation, circadian pattern, and night eating syndrome among older adults, with the path models indicating both direct and indirect associations. These findings emphasize the interconnected nature of seasonal sensitivity, sleep characteristics, circadian preference, and nighttime eating behavior in later life. Further longitudinal research with repeated seasonal assessments is warranted to clarify the temporal relationships among these factors and to determine the potential value of sleep-focused preventive and clinical approaches.

## 1. Introduction

Seasonal variation and population aging have emerged as two of the most critical global health priorities of the twenty-first century. The World Health Organization [1] projects that the population of adults aged 60 years and older will reach 2.1 billion by 2050, fundamentally reshaping healthcare delivery systems and necessitating a paradigm shift in how we approach age-related health conditions influenced by seasonal environmental changes. Seasonal variation represents a fundamental biological reality that manifests as significant fluctuations in mood, sleep patterns, appetite, energy levels, and social engagement across different times of the year [2]. While these seasonal effects are universal human experiences, the intensity of individual sensitivity varies profoundly, with older adults representing a particularly vulnerable population due to age-related physiological changes and reduced adaptive capacity [3,4].

The neurobiological mechanisms underlying seasonal adaptation involve complex interactions between photoperiod detection, circadian entrainment, and neuroendocrine signaling pathways [5]. Seasonal changes influence multiple biological systems, including neurotransmission [6], immune responses [7], gene expression [8], and neuroendocrine function [5]. The suprachiasmatic nucleus, serving as the master circadian pacemaker, receives photic input from intrinsically photosensitive retinal ganglion cells, which are particularly sensitive to short-wavelength blue light [9]. This light-dark cycle information is transduced into rhythmic neuroendocrine signals, primarily through the pineal secretion of melatonin, which encodes night length and season duration [5]. With advancing age, retinal sensitivity diminishes, lens opacification increases, and suprachiasmatic nucleus neuron loss occurs, collectively impairing photic entrainment and reducing the amplitude of circadian output signals [9].

Recent actigraphy-based research has provided robust evidence of age-dependent rest-activity phenotype changes across the human lifespan [10]. Using unsupervised machine learning clustering of minute-level actigraphy profiles, researchers identified that younger individuals exhibit delayed chronotypes with later sleep onset and wake times, whereas older adults demonstrate advanced and more structured activity schedules [10]. These findings establish that aging is associated with fundamental shifts in sleep–wake cycle regulation and activity pattern organization, reflecting both biological changes and behavioral adaptations that increase susceptibility to seasonal environmental fluctuations. The impact of seasonal changes on sleep quality in older adults has been rigorously documented, with studies demonstrating that sunlight intensity has a significant positive effect on time in bed, while humidity and precipitation show negative associations with sleep duration and quality [11].

Sleep quality represents a critical determinant of physical, cognitive, and psychological health in later life [12]. Sleep quality encompasses multiple dimensions, including total sleep time, sleep maintenance efficiency, sleep onset latency, wake after sleep onset, and the frequency of sleep-disruptive events [13]. Poor sleep quality in older adults has been associated with a range of adverse health outcomes, including cardiometabolic risks such as hypertension and insulin resistance [14,15], depressive symptoms [16], and cognitive decline [17]. Furthermore, disrupted circadian rhythms exacerbate sleep problems and contribute to systemic inflammation and metabolic dysregulation [15,18]. Seasonal light exposure represents one of the most potent environmental modulators of sleep quality [19]. Research conducted on elderly populations demonstrated that inter-daily stability of the sleep–wake cycle was significantly higher in autumn and winter, with longer time in bed during these months, and these seasonal variations were negatively linked to ambient temperature [20].

Individual entrainment traits vary significantly across the population, dictating distinct behavioral rhythms and activity timing preferences that define an individual’s circadian pattern or chronotype [21,22]. Circadian patterns are synchronized by internal oscillators and external cues, with light representing the most potent zeitgeber for entrainment [23,24]. Population-level studies have conclusively demonstrated that aging is associated with a phase advance in circadian timing, with older adults exhibiting significantly earlier sleep onset and wake times compared with younger age groups [10]. While most older adults demonstrate a morning circadian preference, a substantial minority maintain an evening chronotype, which has been linked to poorer mood [25], reduced cognitive performance [26], lower health-related quality of life [27], and increased metabolic dysfunction [28]. The relationship between chronotype and seasonal variation has yielded inconsistent findings, with some evidence suggesting chronotypes shift across seasons while other studies find no significant seasonal changes after controlling for light exposure patterns [29,30].

Circadian and seasonal factors can profoundly affect the eating behaviors of older adults [31,32]. Night Eating Syndrome is characterized by three core diagnostic features: morning anorexia, evening hyperphagia with consumption of at least 25% of daily caloric intake after the evening meal, and nocturnal eating episodes occurring at least twice weekly [33,34]. Night eating disrupts circadian alignment [35], increases the risk of metabolic dysfunction [36], and contributes to sleep disturbances [37]. Recent research has clarified that individuals with Night Eating Syndrome tend toward a morning chronotype, suggesting that the syndrome may represent a phase-delayed eating pattern occurring within an otherwise morning-oriented circadian system, creating internal desynchrony between food intake timing and circadian rhythms of appetite-regulating hormones [38,39]. A comprehensive review concluded that Night Eating Syndrome likely has negative impacts on body mass index, diabetes risk, and sleep quality, which in turn may increase cardiometabolic risk [40]. The association between Night Eating Syndrome and sleep quality is well-documented, as nocturnal eating episodes disrupt sleep continuity while poor sleep quality may increase vulnerability to night eating through effects on appetite-regulating hormones, including leptin and ghrelin [41,42,43].

While individual associations among seasonal variation, chronotype, and night eating syndrome have been explored in previous research [3,44,45,46], few studies have systematically examined the mediating role of sleep quality in these relationships. Sağlam et al. [47] investigated seasonality, night eating, and chronotype in bipolar disorder and found that sleep quality partially mediated these associations, suggesting that seasonal effects on eating behaviors operate at least partly through disruption of sleep. However, no study has integrated these factors into a comprehensive mediation model specifically examining community-dwelling older adults. The conceptual framework of the present study operationalizes seasonal variation as the independent variable, with circadian patterns and night eating syndrome as dependent variables and sleep quality as the pivotal mediating mechanism. Egypt provides an appropriate setting for this investigation due to its substantial seasonal variation in daylight duration and temperature across the year [48]. To address the identified gaps, this study aimed to establish a mediation model investigating the direct and indirect effects of seasonal variation on circadian patterns and night eating syndrome through the mediating role of sleep quality among community-dwelling older adults in Egypt. The following research hypotheses were formulated: there is a positive direct association between seasonal variation and both night eating syndrome and evening circadian pattern (H1); there is a negative direct association between seasonal variation and sleep quality (H2); sleep quality serves as a mediating variable in the association between seasonal variation and night eating syndrome (H3); and sleep quality serves as a mediating variable in the association between seasonal variation and circadian pattern (H4).

## 2. Methods

### 2.1. Research Design

This study employed a cross-sectional design conducted in accordance with the Strengthening the Reporting of Observational Studies in Epidemiology (STROBE) guidelines, with data collected between 10 June and 15 September 2025 [49].

**Setting**

The study was conducted in social clubs for older adults operating under the supervision of the Egyptian Ministry of Social Solidarity, which serve individuals from diverse socioeconomic backgrounds by providing social, recreational, cultural, and religious services, including organized trips, celebrations, musical events, and group activities. These settings facilitate regular social interaction and support the well-being of community-dwelling older adults.

**Participants and Eligibility**

**Figure 1:**
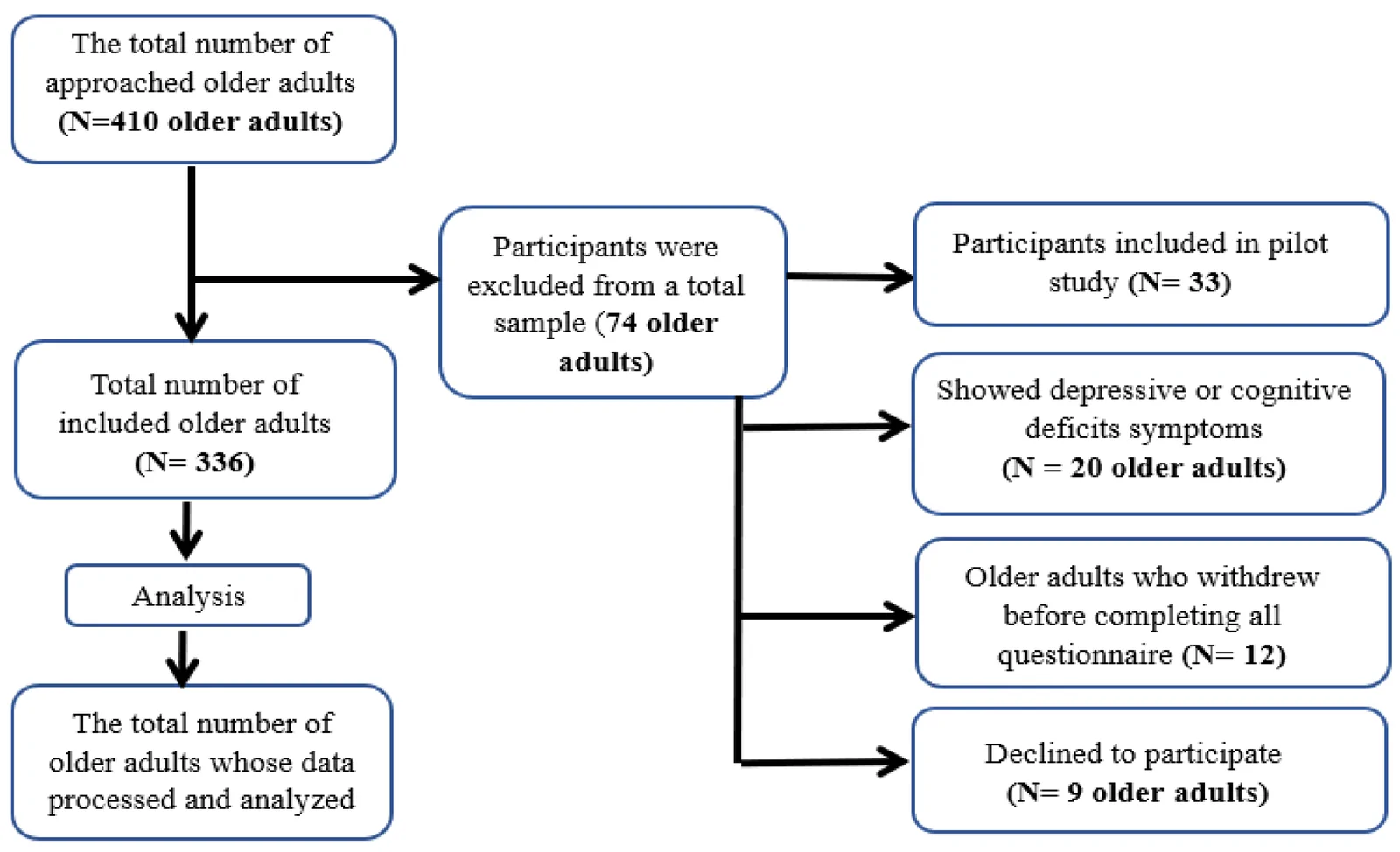
Recruitment flow chart for the studied older adults.

The target population comprised community-dwelling older adults who were active members of government-affiliated geriatric social clubs. Eligible participants were required to be aged 60 years or older, able to communicate in Arabic and understand the study questions with or without interviewer assistance, capable of providing informed consent, and regular members of a geriatric social club. To ensure the validity of responses, all participants underwent cognitive and psychological screening using the Mini-Cog for cognitive function and the Patient Health Questionnaire-9 (PHQ-9) for depressive symptoms. Participants who met the predefined criteria for possible cognitive impairment or clinically relevant depressive symptoms were excluded from the main study (Figure 1).

### 2.2. Sample Size Calculation

Sample size was estimated a priori using the MedPower (8.2.1) interactive software tool for mediation analysis. The following parameters were specified: a desired statistical power of 0.80, a two-tailed alpha level (α) of 0.05, and a conservative moderate effect size (β = 0.30) for all hypothesized pathways in the mediation model, including the path from the independent variable (seasonal variation) to the mediator (sleep quality), from the mediator to the dependent variables (circadian pattern and night eating syndrome), and the direct path from the independent variable to the dependent variables. Based on these parameters, the minimum required sample size was calculated to be 301 participants. To account for an anticipated unresponsive rate of 20%, the sample was inflated accordingly, yielding a target recruitment of 361 participants.

**Recruitment process**

A simple random sampling technique was used. Lists of registered older adults were obtained from the directors of participating geriatric clubs and served as the sampling frame. Using Research Randomizer version 4.0, equal selection probabilities were ensured for individuals on the lists. A total of 410 older adults were initially approached. Of these, 33 participated in the pilot study and were not included in the main study. Among the remaining 377 individuals considered for the main study, 20 were excluded following screening, including eight with clinically relevant depressive symptoms and 12 with possible cognitive impairment. This resulted in 357 eligible participants. Subsequently, 12 participants withdrew before completing the questionnaires and nine declined participation. Therefore, 336 participants completed the main study and were included in the analysis. The completion rate among eligible participants was 94.1% (336/357), while 5.9% (21/357) declined or withdrew (Figure 1).

**Measures of Data Collection**

**Screening Tools**

Cognitive function was assessed using the Arabic version of the Mini-Cog [50], which combines a three-word recall test (0–3 points) and a clock-drawing task (0–2 points). A total score below 3 indicated possible cognitive impairment. The previously published Arabic version demonstrated good internal consistency (Cronbach’s α = 0.89) in an Egyptian older adult population [51].

Depressive symptoms were screened using the Arabic version of the Patient Health Questionnaire-9 (PHQ-9) [52]. This nine-item self-administered instrument assesses symptom frequency over the past two weeks on a 0–3 Likert scale, yielding a total score from 0 to 27, with cutoff scores of 5, 10, and 20 indicating mild, moderate, and severe depression, respectively. The Arabic version has acceptable reliability (Cronbach’s α = 0.88) among Egyptian older adults [52].

**Global Seasonality Scale (GSS)**

The GSS is a core component of the Seasonal Pattern Assessment Questionnaire [53]. It evaluates seasonal variations in six domains: social activity, sleep duration, mood, appetite, weight, and energy level. The instrument utilizes a weighted scoring system (0–4) to quantify fluctuations. With a total score range of 0 to 24, the scale provides a robust metric for assessing the magnitude of an individual’s seasonal variation. Higher scores reflect greater seasonal variation. The study utilized validated thresholds to segment participants by seasonal sensitivity: low (≤7), moderate (8–10), and high (≥11). These categories ensure a precise comparison between different levels of variation. Because the month of questionnaire completion may influence the GSS responses, the assessment was adapted by asking participants to report their seasonal patterns throughout the year, indicating the months in which they experienced their best and worst sleep and appetite quality. The year was divided into four seasons for simplicity: summer (May–July), autumn (August–October), winter (November–January), and spring (February–April). In the current study, the GSS demonstrated strong internal consistency (Cronbach’s α = 0.911).

**Morningness–Eveningness Questionnaire (MEQ)**

The MEQ is a 19-item self-report questionnaire that assesses preferred sleep-awake times, physical and mental performance periods, and optimal times for daily activities [22]. Items use 4–5-point Likert-type scales to measure circadian preference. Composite scores range from 16 to 86, with lower scores indicating eveningness and higher scores indicating morningness. Participants were mapped to five validated circadian patterns: Definite Morning (70–86), Moderate Morning (59–69), Neutral (42–58), Moderate Evening (31–41), and Definite Evening (16–30). In this study, the MEQ demonstrated excellent internal consistency (Cronbach’s α = 0.94).

**Night Eating Diagnostic Questionnaire (NEDQ)**

The NEDQ is a 22-item measure for diagnosing NES [54]. It includes a checklist of symptoms related to nighttime food intake, sleep-eating patterns, awareness of nocturnal eating, and associated distress. The items were mainly dichotomous (yes/no) or open-ended (e.g., percentage of daily intake consumed after the evening meal). The NEDQ evaluates six diagnostic criteria for NES based on the latest guidelines [33], without providing a single numerical score. A hierarchical scoring method categorizes respondents as absent (0), mild (1), moderate (2), or full NES (3) [42,55]. In this study, the NEDQ items demonstrated high internal consistency (Cronbach’s α = 0.89).

**Pittsburgh Sleep Quality Index (PSQI)**

The PSQI is a 19-item self-rated measure that evaluates SQ over the past month across seven components: subjective SQ, sleep latency, sleep duration, sleep disturbances, habitual sleep efficiency, use of sleep medication, and daytime dysfunction [56]. Each Component is scored from 0 (no difficulty) to 3 (severe difficulty), with a global score ranging from 0 to 21. Higher scores indicate a poorer SQ. A score > 5 indicates poor SQ, whereas a score ≤ 5 indicates good SQ. In the current study, the PSQI demonstrated acceptable internal consistency (Cronbach’s α = 0.73).

**Sociodemographic and Clinical Data Sheet**

Sociodemographic and clinical sheets included questions about age, gender, occupation (current and previous), residence, marital status, education level, monthly income, and current health conditions. Anthropometric measurements (weight and height) were performed, and the Body Mass Index (BMI) was calculated using the formula weight (kg)/height^2^ (m^2^). BMI categories followed the World Health Organization standards: underweight (<18.5), normal (18.5–24.9), overweight (25–29.9), and obese (≥30.0) [57].

## 3. Procedure

**Ethical Considerations**

Ethical approval was obtained from the Research Ethics Committee of the Faculty of Nursing, Zagazig University, Egypt (IRB: NUR 61: 2/6/2025). The study complied with all applicable local laws and regulations and adhered to the ethical principles outlined in the Declaration of Helsinki (DoH, October 2008). All participants received clear information about the study’s purpose and were informed that participation was voluntary and anonymous. Written informed consent was secured from each participant prior to data collection. Participants were assured of their right to withdraw from the study at any time without negative consequences.

**Tool Validity**

To ensure the appropriateness and relevance of the study instruments for the target population, a comprehensive validity assessment was conducted prior to data collection. A panel of five experts in gerontological and community health nursing evaluated the face validity of the Arabic versions of the Global Seasonality Scale (GSS), Morningness–Eveningness Questionnaire (MEQ), Night Eating Diagnostic Questionnaire (NEDQ), and Pittsburgh Sleep Quality Index (PSQI), assessing each instrument for clarity, relevance, comprehensiveness, and cultural appropriateness for Egyptian older adults. Following face validity assessment, the same panel evaluated content validity using a quantitative approach, rating each item on a 4-point relevance scale; the Item-Level Content Validity Index (I-CVI) and Scale-Level Content Validity Index (S-CVI) were calculated, with all instruments achieving S-CVI values above 0.90 (GSS = 0.92, MEQ = 0.94, NEDQ = 0.91, PSQI = 0.93), indicating excellent content validity. Confirmatory factor analysis (CFA) confirmed the hypothesized factor structures for all instruments, with all items loading significantly on their respective factors (factor loadings > 0.50, *p* < 0.001) and acceptable model fit indices (CFI > 0.90, RMSEA < 0.08), establishing robust construct validity for the study population.

**Pilot Study:** A pilot study was conducted with 33 community-dwelling older adults who met the same eligibility criteria as the main study sample but were recruited from geriatric social clubs not included in the main study to avoid contamination. The pilot study assessed the feasibility of study procedures, tested the clarity and comprehensibility of all instruments, estimated the average completion time (22 min, range 18–28 min), and identified any logistical or administrative challenges. Based on participant feedback, minor wording modifications were made to three items in the sociodemographic data sheet to improve clarity, and a standardized clarification script was developed for distinguishing between “appetite” and “hunger” on the GSS. Internal consistency reliability was calculated for each multi-item instrument using Cronbach’s alpha: the GSS demonstrated excellent reliability (α = 0.91), the MEQ demonstrated excellent reliability (α = 0.94), the NEDQ demonstrated good reliability (α = 0.89), and the PSQI demonstrated acceptable reliability (α = 0.73), confirming that all instruments performed adequately in the Egyptian older adult population. The 33 pilot study participants were excluded from the main study sample to prevent contamination, as recommended by best practices for pilot study methodology.

### 3.1. Data Collection

Data were collected between 10 June and 15 September 2025, through standardized face-to-face interviews conducted by a trained researcher in designated quiet and private areas within the participating geriatric social clubs. Before data collection, potentially eligible older adults received a clear explanation of the study objectives, procedures, expected duration, voluntary nature of participation, and their right to decline participation or withdraw at any time without any consequences. Written informed consent was obtained before screening and questionnaire administration. Participants first underwent cognitive and depressive-symptom screening using the Mini-Cog and PHQ-9, respectively. Individuals who met the predefined exclusion criteria were not enrolled in the main study. For eligible participants, the researcher subsequently administered the sociodemographic questionnaire, GSS, MEQ, NEDQ, and PSQI. The Mini-Cog was administered according to its standardized procedure, whereas the remaining instruments were administered verbally using a standardized interviewer-assisted format. Each item and its response options were read aloud in a neutral and consistent manner, and participants’ responses were recorded by the interviewer. This procedure was applied consistently to all participants and enabled the inclusion of older adults with different educational and literacy levels.

Each interview lasted approximately 20–25 min. Interviews were conducted individually to minimize interruptions and protect participants’ privacy. Participants were encouraged to request clarification when needed; however, the researcher avoided interpreting questions in a manner that could influence participants’ responses. A standardized explanation was used whenever clarification was required to maintain consistency across interviews. To protect participants’ confidentiality, questionnaires were identified using unique study codes rather than participants’ names or other directly identifying information. Immediately after each interview, the researcher reviewed the questionnaire for completeness and internal consistency to minimize missing or incomplete responses. Completed questionnaires were then securely stored until data entry and analysis. Data were entered into the statistical database using the assigned study codes. Electronic data were maintained in a password-protected file accessible only to authorized members of the research team.

### 3.2. Data Processing and Mediation Analysis

Data were entered and analyzed using IBM SPSS Statistics version 23.0. Qualitative variables were summarized as frequencies and percentages, whereas quantitative data were presented as means, standard deviations, medians, and ranges (minimum–maximum), as appropriate. Comparisons between categorical variables were performed using the chi-square test. Pearson’s correlation coefficient was used to assess the bivariate relationships among the main study variables. Multiple linear regression analyses quantified the predictive relationships between the independent and dependent variables. By isolating these factors, the models established the strength and direction of each association. Multicollinearity was assessed using the Variance Inflation Factor (VIF), with values above 4 or tolerance values below 0.25 indicating no multicollinearity concerns. All statistical tests were two-tailed, and statistical significance was set at *p* < 0.05. Path analysis, a form of structural equation modeling, was employed to examine the hypothesized relationships among seasonal variation, sleep quality, circadian patterns, and night eating syndrome. By integrating simultaneous estimation of direct and indirect pathways, this method isolates the mediating role of sleep quality, providing a clear lens through which to evaluate complex causal mechanisms. Residual variances, represented by the curved arrows in the model, indicate the proportion of unexplained variance in each endogenous variable.

Mediation analyses were conducted using JASP version 0.16. A bootstrapping approach with 5000 resamples was used. Bootstrapping is a non-parametric method that estimates the sampling distribution of indirect effects and provides bias-corrected 95% confidence intervals. Mediation was considered statistically significant when the confidence interval did not include zero. Calculating the proportion of the total effect validated sleep quality as a key mediator, revealing the specific magnitude of its influence within the model.

## 4. Results

The sample consisted of 336 participants, of whom 191 (56.8%) were men and 145 (43.2%) were women. The mean age was 66.8 ± 5.9 years (range, 60–87 years). Further demographic details are shown in Table 1. Data analysis reveals compelling correlations between seasonal variation scores and key demographic factors. Seasonal-variation categories were significantly associated with age (*p* = 0.048), sex (*p* = 0.002), BMI (*p* < 0.001), residence (*p* < 0.001), previous employment status (*p* < 0.001), and current employment status (*p* = 0.001), as shown in Table 1.

Moderate-to-severe seasonal variation was observed in 68.2% of participants, and the mean GSS score was 9.4 ± 5.3. A morning circadian preference, combining moderate and definite morning patterns, was observed in 56.9% of participants, whereas 27.4% demonstrated moderate or definite evening patterns and 15.8% had an intermediate pattern. Regarding night eating, 26.8% had no NES, 46.4% were classified as mild night eaters, 3.6% as moderate night eaters, and 23.2% met the criteria for full NES. Thus, 73.2% exhibited some level of night-eating symptoms, ranging from mild symptoms to full syndrome. Poor sleep quality was reported by 85.1% of participants, with a mean global PSQI score of 9.1 ± 3.5 (Table 2).

**Figure 2:**
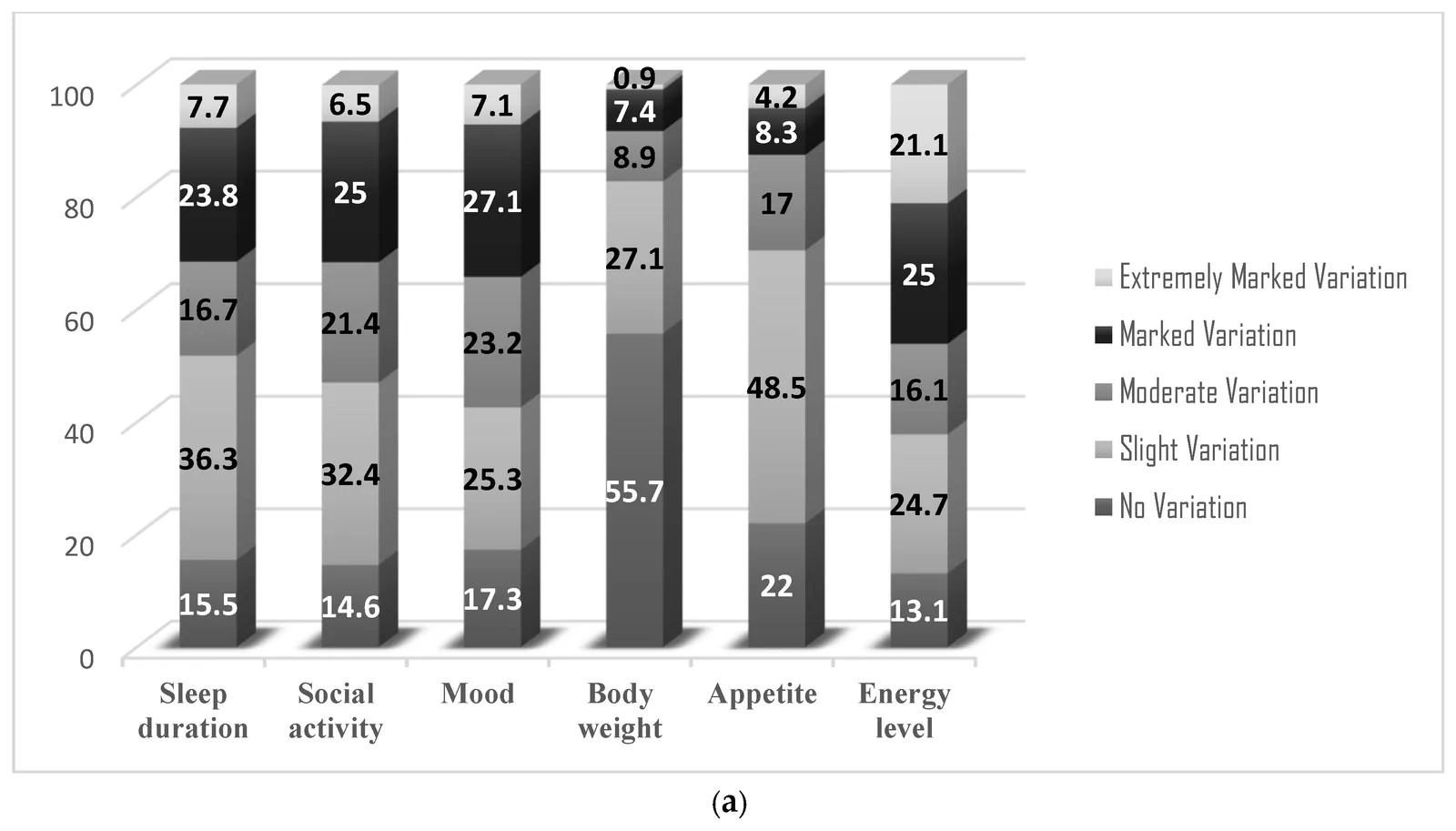

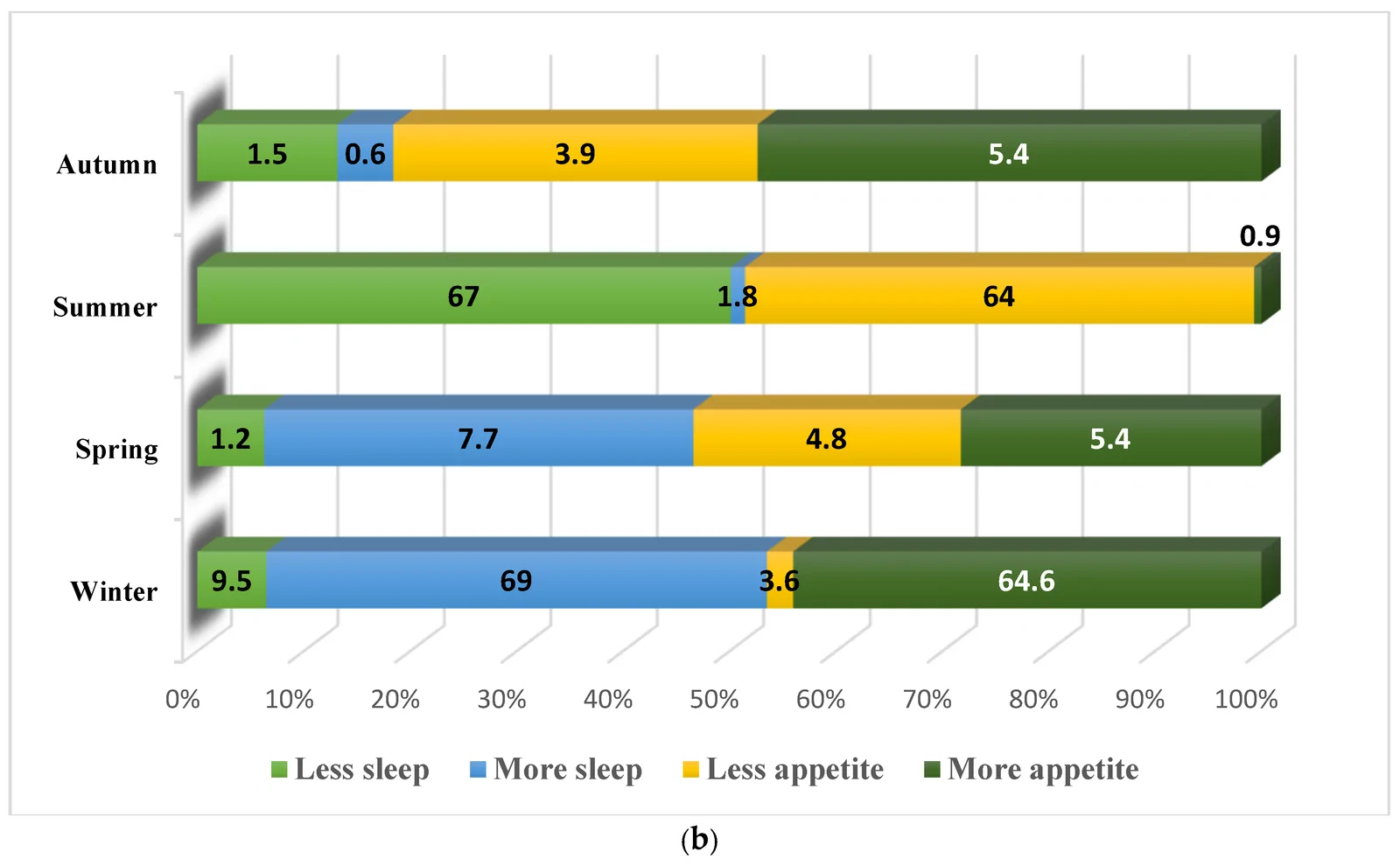
Distribution of variation levels among participants (N = 336) (**a**) according to their global seasonality score (**b**) across four seasons of the year in sleep and appetite.

**Figure 3:**
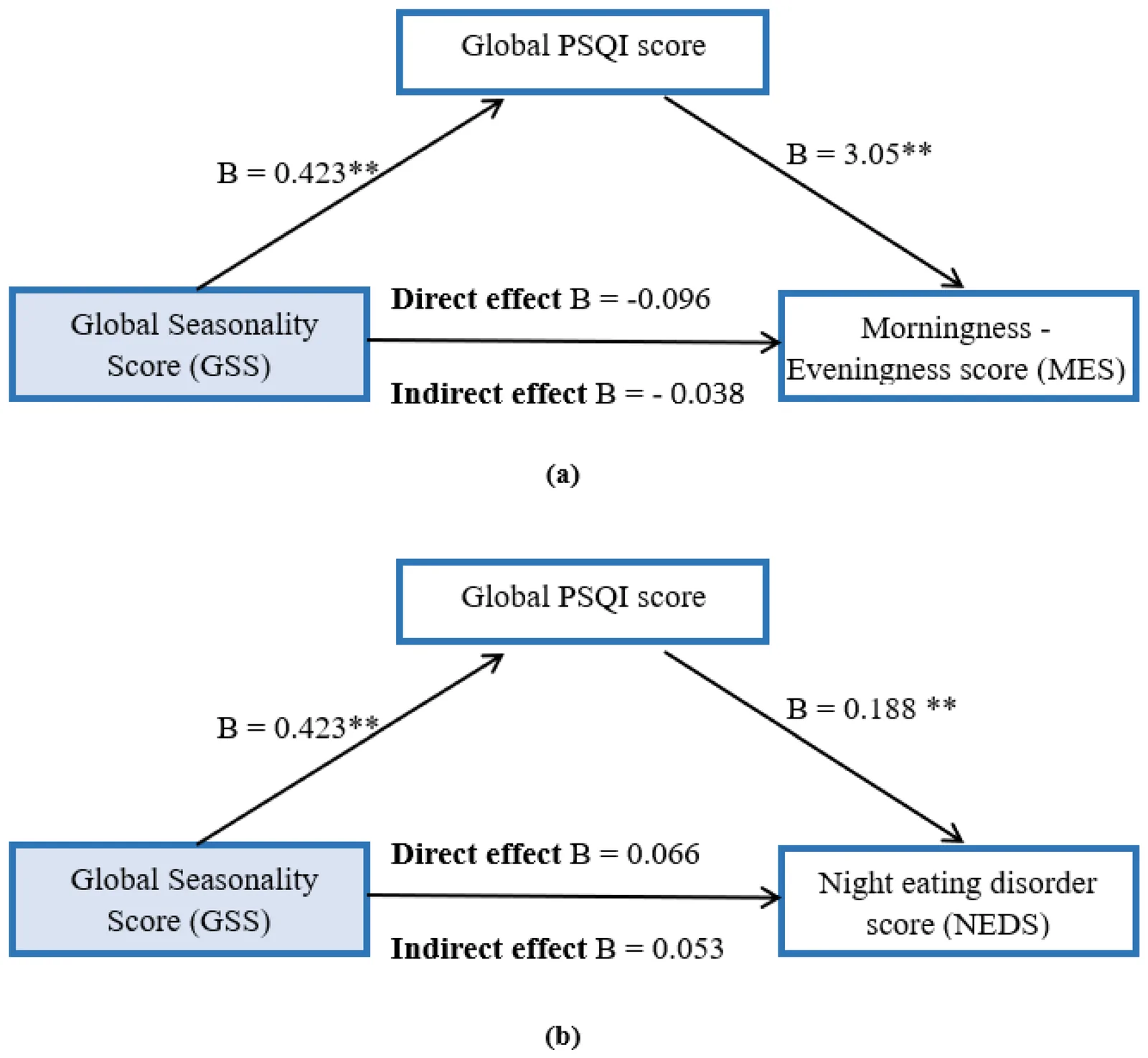
The path analyses of (**a**) the non-standardized coefficients assessed SV’s direct and indirect effect on circadian patterns, with SQ score as a mediating factor (** *p* < 0.001). (**b**) The unstandardized coefficients assessed SV’s direct and indirect effect on NES, with SQ as a mediating factor (** *p* < 0.001).

Figure 2 displays the distribution of seasonal variation levels among participants; 68.2% of older adults experienced moderate to severe seasonal variation, with a mean GSS score of 9.4 (SD = 5.3). Participants reported slight to moderate seasonal fluctuations across sleep duration, social activity, mood, weight, appetite, and energy. Appetite changes were reported by 48.5% of older adults, while 27.1% noted seasonal mood variations. Conspicuously, Figure 3b indicates that most participants reported increased sleep duration and greater food intake during winter (69% and 64.6%, respectively). Conversely, summer was associated with sleep difficulties (67%) and reduced food intake (64%).

The correlation coefficient in Table 3 demonstrates robust positive relationships across all key variables. Most notably, seasonal variation correlates strongly with sleep quality (r = 0.638, *p* < 0.001). Furthermore, Night Eating Syndrome showed highly significant associations with both SV (r = 0.581, *p* < 0.001) and SQ (r = 0.609, *p* < 0.001), confirming a tight interlinking of these behaviors. Older adults with NES exhibited greater seasonal variation, poorer sleep quality, and stronger eveningness. Correlation analysis confirms that circadian patterns are intrinsically tied to seasonal and eating behaviors. The significant negative coefficients (all r > −0.64, *p* < 0.001) underscore a clear inverse dependency between CP and the participants’ SV, SQ, and NES profiles. Older adults with an evening circadian pattern experienced greater SV, poorer SQ, and exhibited night eating syndrome.

Referring to the multivariate analysis of MES and NES scores, Table 4 indicates that the night eating and global seasonality score were statistically significant independent positive predictors of MES score (*p* = 0.0001). Referring to the multivariate analysis of the NES score, Table 4 indicates that MES, BMI, and PSQI were statistically significant positive predictors of the NES score (*p* = 0.000). In particular, night eating predicts later circadian pattern, higher body mass index, and poor sleep quality, while the seasonal variation predicts night eating and later circadian pattern.

**Figure 4:**
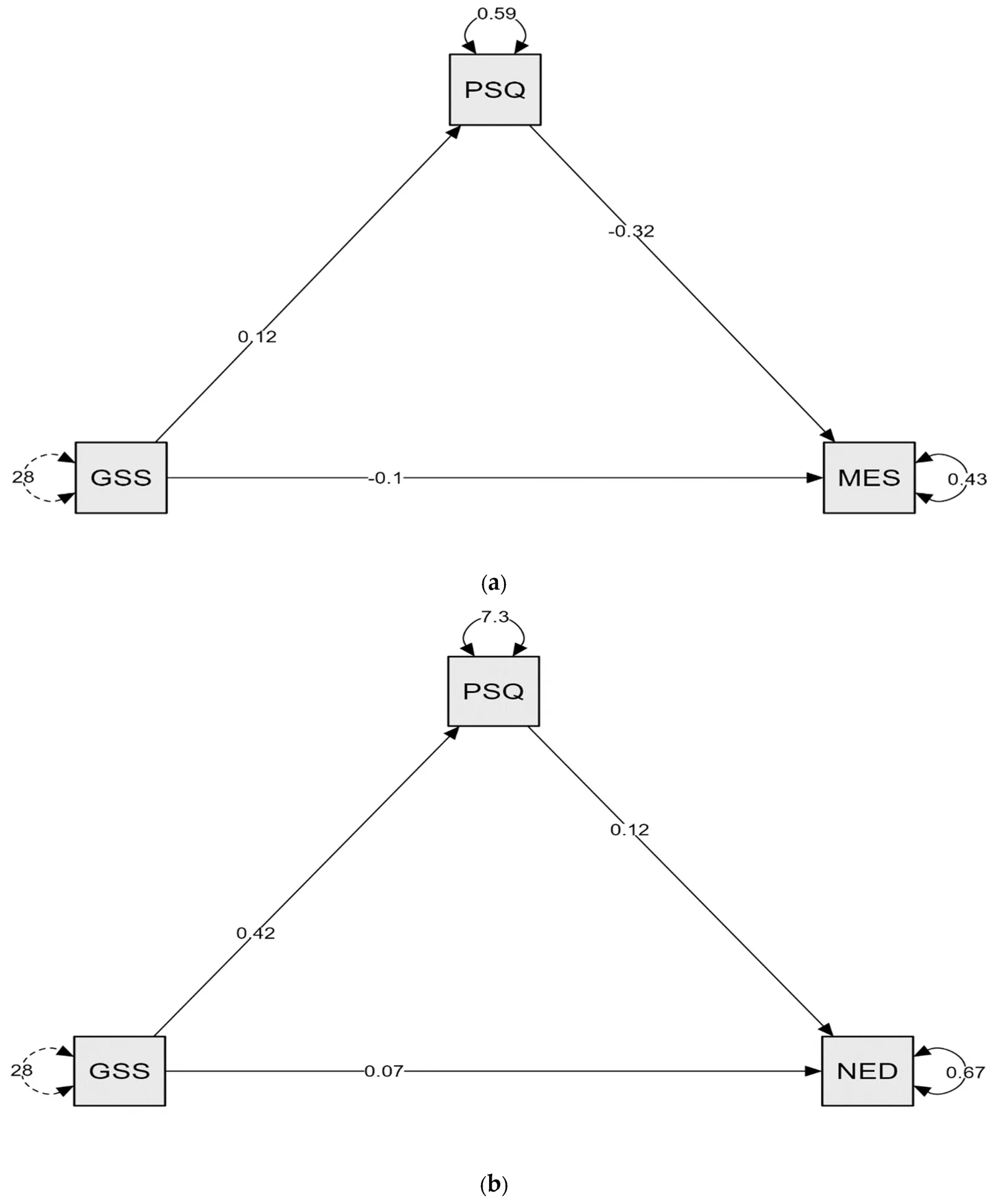
Path plot models of (**a**) GSS predicting MES, mediated by global PSQI; (**b**) GSS predicting NES, mediated by global PSQI.

The bootstrap analysis results, synthesized in Figure 3 and Table 5, substantiate the significance of our findings. The data demonstrate a high degree of confidence in the estimated parameters. As shown in Figure 4a and Table 5, SV significantly predicted global PSQI scores (B = 0.423, z = 15.16, *p* < 0.001), and the global PSQI score significantly predicted the MES score (B = 3.05, z = 15.3, *p* < 0.001). The indirect effect of SV on circadian patterns through the PSQI was significant (B = −0.038, z = 6.213, *p* < 0.001), as was the direct effect after controlling for the PSQI (B = −0.096, z = 6.213, *p* < 0.001). Sleep quality accounted for 41% of the total effect, indicating a partial mediation. Similarly, Figure 4b and Table 5 show that SV significantly predicted both PSQI (B = 0.423, z = 15.16, *p* < 0.001) and NES (B = 0.188, z = 14.03, *p* < 0.001). The indirect effect of SV on NES through PSQI was significant (B = 0.053, z = 6.75, *p* < 0.001), and the direct effect remained significant after controlling for PSQI (B = 0.066, z = 6.75, *p* < 0.001). SQ explained 37% of the total effect, indicating partial mediation.

Finally, Figure 4 and Table 6 provide the standardized path coefficients from the GSS predicting MES and NES through PSQI, supported by bootstrap estimates (5000 samples) with associated confidence intervals.

## 5. Discussion

This study investigated the interrelationships among seasonal variation, circadian pattern, night eating syndrome, and sleep quality in community-dwelling older adults, with particular attention to the potential intermediary role of sleep quality. The hypothesized path models identified statistically significant direct and indirect associations among the study variables, suggesting that sleep quality may contribute meaningfully to the observed relationships between seasonality, circadian preference, and night eating behavior. These mediation estimates should be interpreted as model-based associations within a cross-sectional framework rather than as evidence of temporal or causal pathways. To the best of our knowledge, this study is among the first to examine these interrelated factors concurrently and to evaluate the potential mediating role of sleep quality in an older Egyptian population.

### 5.1. Seasonal Variation, Circadian Patterns, NES, and SQ

The results indicated that most participants experienced moderate to severe SV levels. This finding aligns with prior evidence suggesting that seasonal variation may intensify with aging due to physiological and psychosocial changes associated with circadian rhythm regulation. Shoukr et al. reported similar findings in Egypt, observing that many older adults experience seasonal fluctuations in sleep duration, mood, weight, social activity, appetite, and energy levels [52]. These results are consistent with those of earlier studies that identified high global seasonality scores among older adults [58].

However, the current results contrast with the findings of Holler et al. in Iceland, who reported that younger age groups exhibited greater seasonality. This discrepancy may be attributed to the cultural and environmental factors that influence individuals’ perceptions of seasonal changes. For instance, older generations in some regions may receive winter’s darkness more positively and maintain stable routines that buffer seasonal mood changes, whereas younger individuals may be more affected by lifestyle disruptions related to variations in daylight [3,10,59].

Consistent with previous research, participants in the present study reported a pattern of longer sleep duration during winter than during summer. This seasonal difference may be related to changes in daylight exposure and ambient temperature, as suggested by Hashizaki et al. [60] and Yetish et al. [61]. Similarly, Guo et al. [11] reported poorer sleep characteristics during periods of higher summer temperatures. These findings support the observed seasonal pattern in sleep reported by our participants. However, because seasonal information in the present study was obtained retrospectively rather than through repeated assessments across seasons, these findings should be interpreted as perceived seasonal differences rather than prospectively observed within-person changes.

The morning pattern was predominant among the participants. Several contextual and sociocultural factors may explain this trend. Many older adults in Egypt, particularly those from rural settings, have a long-standing history of agrarian work that requires early wake times, which may reinforce morning-oriented sleep patterns [62]. Furthermore, older adults generally have lower exposure to digital devices than younger generations, resulting in reduced exposure to evening blue light, a factor known to suppress melatonin secretion and promote evening alertness [63]. Their rural background and minimal technological footprint directly drive a stronger morning preference. This lifestyle reinforces natural light-dark synchronization, minimizing the circadian delays typically caused by artificial light.

A morning circadian preference predominated in the present sample, a finding that is consistent with previous studies demonstrating a progressive shift toward earlier sleep and wake timing with advancing age [9,45,64]. Age-related physiological changes, including reduced retinal sensitivity and alterations in suprachiasmatic nucleus signaling, may contribute to this advancement in circadian phase, while retirement may permit sleep–wake schedules to align more closely with intrinsic circadian preference [3,64]. Contextual factors such as occupational routines, exposure to natural daylight, and lower evening exposure to artificial or blue light have also been proposed as potential contributors to morningness in older populations [62]. However, these factors were not directly assessed in the present study and should therefore be considered plausible explanations based on previous literature rather than mechanisms established by the current findings.

Age-related physiological changes may have also contributed to this shift. Diminished retinal sensitivity and weakened suprachiasmatic nucleus signaling directly impair photic entrainment. This impairment shifts the internal clock forward, mandating the advanced circadian phases and earlier sleep–wake cycles typical of older age. Höller et al. [3] demonstrated that older adults, especially retirees, tend to revert to sleep–wake patterns that more closely align with their intrinsic circadian preferences.

The prevalence of NES in this study was notably higher than that reported in many other regions, including Germany (1.1%) [65], Japan (1.5%) [66], Australia (0.9%) [67], urkey (10%) [68], and Pakistan (14.4%) [69]. Blouchou et al. also found that 8.1% of participants exhibited NES symptoms in their study. Several factors may explain the higher prevalence observed in this study. First, the sample consisted exclusively of adults aged 60 years and above, an age group that is more vulnerable to circadian rhythm disruption and sleep problems. Second, most participants experienced poor SQ, which has been linked to increased nocturnal eating as a coping or compensatory behavior. Recent evidence also suggests that irregular circadian rhythms may delay meal timing and increase the consumption of energy-dense foods, supporting the hypothesis that an evening pattern may predispose individuals to NES [38,39]

With respect to SQ, the majority of participants reported poor sleep, which may be explained by age-related reductions in melatonin production, increased nocturnal awakenings, reduced slow-wave sleep, and the presence of physical and psychological comorbidities. Prior research indicates that older adults often experience insufficient exposure to daytime light, which further compromises circadian regulation [20]. Rezaei et al. found that over half of their participants reported poor SQ (mean PSQI = 6.32) [70]. Similarly, Patel et al. concluded that sleep disturbances increase markedly with age, affecting up to 50% of older adults [71]. Miner and Kryger further noted that 33–66% of older adults report sleep complaints, depending on the comorbidity burden [72].

### 5.2. Correlations and Factors

Pearson’s correlation analysis revealed strong associations between circadian patterns, SV, and SQ. Alterations in melatonin circadian profiles support the premise that circadian patterns significantly influence SV [29]. Differences in environmental or genetically mediated light sensitivity may also affect circadian phase shifts, particularly during seasonal changes in daylight [30]. Evening exposure to artificial light can further contribute to circadian phase delays and heightened mood seasonality. As urbanization increases, with more time spent indoors and reduced exposure to natural daylight, the human connection to natural seasonal cues may progressively weaken.

This study revealed a significant trend of NES-positive screening among overweight and obese older adults compared with those of normal weight (*p* = 0.0001). The timing and nutritional composition of food intake act as essential zeitgebers for the human biological clock, supporting previous research findings [38]). In contrast, research from Saudi Arabia indicates that BMI remains consistent regardless of NES status, showing no statistically significant differences between groups. This inconsistency reflects the variability in NES prevalence across older populations, which is influenced by cultural factors, lifestyle habits, and differences in sample characteristics [73].

Consistent with Nolan et al., this study found higher PSQI scores in participants with moderate or full NES symptoms than in those with mild or no symptoms [42]. Similarly, Abo El-Ela et al. reported a significant association between increasing NEDQ symptoms and poorer SQ, (Abo El-Ela et al., [74], while Guo et al. in China found no relation between sleep disorders and night eating scores [11].

The nexus of NES, sleep, and obesity is driven by an interdependent framework of behavioral, hormonal, and psychological mechanisms. These pathways do not act in isolation; they converge to dictate metabolic and behavioral outcomes. NES, characterized by delayed eating patterns, disrupts hormonal regulation, particularly leptin and ghrelin, which affect appetite, weight control, and metabolic function, potentially contributing to sleep disturbances [41,43]. These results align with prior studies indicating that individuals with NES commonly experience weight gain, obesity, poor SQ, and recurrent nocturnal eating [40,68]. Sayed Ahmed et al. documented a significant association between NES and sleep disturbances among obese adults [43].

A significant positive correlation was observed between circadian patterns and SQ, with evening-type individuals reporting more frequent sleep problems than morning and intermediate types. Evening patterns often exhibit irregular sleep–wake patterns and increased daytime napping, which contribute to greater sleep disruptions and cumulative sleep debt [45]. This is consistent with prior research demonstrating that a later sleep midpoint is associated with poorer sleep outcomes. Bodur et al. further confirmed that evening-type individuals experience significantly poorer SQ than other chronotypes (*p* = 0.009 and <0.001, respectively) [75].

### 5.3. Seasonal Variation and CPs Through SQ as a Mediator

Path analysis indicated a significant indirect association between seasonal variation and circadian pattern through sleep quality, suggesting that sleep quality may represent an important intermediary component in this relationship. This finding is consistent with previous studies reporting that greater eveningness is associated with higher seasonality scores and poorer sleep characteristics [3,9]. Seasonal differences in sleep duration and quality have also been documented, with shorter sleep duration and reduced sleep efficiency reported during warmer periods and under varying environmental conditions [11]. In addition, variation in light exposure and melatonin circadian profiles has been associated with both seasonal sensitivity and circadian timing [29]. Indoor environmental factors, including CO_2_ concentration, have likewise been linked to sleep quality in previous research [75]. Collectively, these findings support the plausibility of sleep quality as an intermediary factor linking seasonal variation with circadian preference. However, because environmental exposures were not directly measured in the present study and the mediation model was based on cross-sectional data, these factors should be regarded as contextual explanations rather than mechanisms established by the current analysis.

Furthermore, several studies have demonstrated that individuals with greater eveningness tend to report higher seasonality scores [9]. This association has also been examined in relation to evening light exposure, day length, and melatonin circadian profiles [9]. In the present study, the significant indirect pathway through sleep quality suggests that sleep may contribute to the observed relationship between seasonal variation and circadian preference. Nevertheless, the temporal sequence of these relationships cannot be established from the current cross-sectional model and warrants confirmation in longitudinal studies.

### 5.4. Seasonal Variation and NES Through SQ as a Mediator

Path analysis identified a statistically significant indirect association between seasonal variation and circadian pattern through sleep quality, supporting a partial mediation pattern within the hypothesized model. This finding is consistent with previous studies reporting that greater eveningness is associated with higher seasonality and poorer sleep characteristics [3,9]. Related evidence has also linked seasonal differences in light exposure and melatonin circadian profiles with variations in circadian timing and sleep regulation [29]. Collectively, these findings support the relevance of sleep quality in the relationship between seasonal variation and circadian preference. However, given the cross-sectional design, the temporal sequence of these associations cannot be established, and sleep quality should be interpreted as a statistical intermediary rather than a demonstrated causal mechanism.

**Limitations of the study**

Several limitations should be considered when interpreting the findings of this study. First, the cross-sectional design limits the ability to establish temporal sequencing or causal relationships among seasonal variation, sleep quality, circadian pattern, and night eating syndrome. Accordingly, the direct and indirect effects identified in the path models should be interpreted as statistical associations rather than evidence of causal mediation. Second, data were collected during a single period of the year, and seasonal variation was assessed retrospectively rather than through repeated prospective assessments across the four seasons. This approach may be subject to recall bias and does not permit direct evaluation of within-person seasonal changes over time. Third, the study relied predominantly on self-reported measures, which may introduce reporting and recall biases. Fourth, potentially relevant environmental and behavioral exposures, including ambient temperature, light exposure, indoor climate, CO_2_ concentration, and digital-device exposure, were not directly measured; therefore, their contribution to the observed associations could not be determined. Finally, participants were recruited from geriatric social clubs within a single geographic area, which may limit the generalizability of the findings to older adults in other regions, institutional settings, or populations with different environmental and sociodemographic characteristics. Future longitudinal and interventional studies incorporating repeated seasonal assessments, objective measures, and more diverse populations are warranted to clarify the temporal and contextual nature of these relationships.

## 6. Conclusions

This study demonstrated significant interrelationships among seasonal variation, sleep quality, circadian pattern, and night eating syndrome in community-dwelling older adults. Moderate-to-severe seasonal variation and poor sleep quality were common, while morning circadian preference was the predominant pattern and a notable proportion of participants met the criteria for full night eating syndrome. The path models identified significant direct and indirect associations, with sleep quality emerging as an important intermediary factor in the relationships of seasonal variation with circadian pattern and night eating syndrome. These findings highlight the relevance of considering sleep quality when examining seasonal and circadian influences on eating behavior in later life. However, the observed pathways should be interpreted within the context of the study design and do not establish temporal or causal relationships. Further longitudinal studies incorporating repeated seasonal assessments and objective measures are needed to clarify the direction and stability of these associations and to evaluate their potential clinical and public health relevance.

**Clinical Implications**

The findings of this study suggest that sleep quality may be clinically relevant when evaluating older adults who report marked seasonal variation, circadian preference differences, or night eating symptoms. In routine geriatric assessment, healthcare providers may consider exploring changes in sleep quality, sleep timing, appetite, and nighttime eating behaviors when clinically indicated, particularly among older adults presenting with related concerns. At the same time, the present findings do not provide sufficient evidence to support mandatory seasonal screening, chronotherapeutic intervention, or season-specific treatment protocols. Rather, they highlight potential areas for clinical attention that should be evaluated further in prospective research. Sleep hygiene education, behavioral counseling, and assessment of circadian and eating patterns may warrant consideration as supportive approaches, but their effectiveness in relation to seasonal variation requires confirmation through longitudinal and interventional studies.

## Figures and Tables

**Table 1:** Demographic statistics and influence on seasonal variation.

Variables	Categories	Seasonal Variation (*N* = 336)						*N*	χ ^2^	*p*
		No (*N* = 107)		Moderate (*N* = 102)		Severe (*N* = 127)				
		No.	(%)	No.	(%)	No.	(%)			
Age	≤70 years	93	(86.9)	82	(80.4)	94	(74.0)	269	6.06	0.048
	>70 years	14	(13.1)	20	(19.6)	33	(26.0)	67		
Gender	Male	74	(69.2)	58	(56.9)	59	(46.5)	191	12.2	0.002
	Female	33	(30.8)	44	(43.1)	68	(53.5)	145		
BMI	Under weight	1	(0.9)	3	(2.9)	2	(1.6)	6		
	Normal weight	87	(81.3)	72	(70.6)	55	(43.3)	214	49.3	0.0001
	Overweight	18	(16.8)	22	(21.6)	45	(35.4)	85		
	Obese	1	(0.9)	5	(4.9)	25	(19.7)	31		
Education level	Illiterate	23	(21.5)	21	(20.6)	18	(14.2)	62		
	Read and write	15	(14.0)	14	(13.7)	15	(11.8)	44	9.43	0.49
	Basic/intermediate	16	(14.9)	28	(27.4)	36	(28.4)	80		
	University/Postgraduate	53	(49.5)	39	(38.2)	58	(45.7)	150		
Marital status	Married	87	(81.3)	73	(71.6)	103	(81.1)	263	4.37	0.36
	Divorced	3	(2.8)	4	(3.9)	2	(1.6)	9		
	widow	17	(15.9)	25	(24.5)	22	(17.3)	64		
Residence	Rural	78	(72.9)	68	(66.7)	39	(30.7)	185	49.7	0.0001
	Urban	29	(27.1)	34	(33.3)	88	(69.3)	151		
	Employee	58	(4.2)	44	(43.1)	60	(47.2)	162		
Employment before retirement	Worker	12	(11.2)	16	(15.7)	11	(8.7)	39		
	Unemployed	37	(34.6)	42	(41.1)	56	(44)	135	39.5	0.0001
Income	Not enough	32	(29.9)	33	(32.4)	31	(24.4)	96	7.1	0.13
	Enough	72	(67.3)	64	(62.7)	83	(65.4)	219		
	Enough and saving	3	(2.8)	5	(4.9)	13	(10.2)	21		
Current job	Yes	27	(25.2)	21	(20.6)	9	(7.1)	57	14.9	0.001
	No	80	(74.8)	81	(79.4)	118	(92.9)	279		
Live with	Alone	4	(3.7)	8	(7.8)	9	(7.1)	21	1.75	0.42
	With family	103	(96.3)	94	(92.2)	118	(92.9)	315		

χ ^2^ Chi square test; non-significant *p* > 0.05 *p* < 0.05: significant.

**Table 2:** Descriptive statistics of variables (*N* = 336).

Variables	Categories	No & M (SD)	%
**Seasonal Variation**	No Variation	107	31.8
	Moderate Variation	102	30.4
	Severe Variation	127	37.8
	***Total Seasonal Variation***		
	***M (SD)***	*9.4 (5.3)*	
	***Median (Range)***	*9 (0–24)*	
**Circadian Patterns**	Definite Evening pattern	21	6.3
	Moderate Evening pattern	71	21.1
	Intermediate pattern	53	15.8
	Moderate Morning pattern	102	30.4
	Definite Morning pattern	89	26.5
	***M (SD)***	*56.3 (16.68)*	
	***Min.–Max.***	*17 (84)*	
**Night Eating Syndrome**	Normal	90	26.8
	Mild Night Eater	156	46.4
	Moderate Night Eater	12	3.6
	Full Syndrome Night Eater	78	23.2
**Sleep Quality**	Subjective SQ	1.4 (0.76)	
	Sleep Latency (min)	1.2 ± 0.53	
	Sleep Duration (h)	1.6 ± 0.84	
	Habitual Sleep Efficiency (%)	2.1 ± 1.2	
	Sleep Disturbance	1.3 ± 0.5	
	Use of Sleep Medication	0.04 ± 0.3	
	Daytime Dysfunction	1.5 ± 0.9	
	***Total sleep quality***		
	***Poor Quality (> 5)***	*286*	*85.1*
	***Good Quality (≤5)***	*50*	*14.9*
	***M (SD)***	*9.1 (3.5)*	
	***Min.–Max.***	*0 (16)*	

**Table 3:** Correlation analysis of variables (N = 336).

Correlations		Seasonal Variation	Sleep Quality	Circadian Patterns	Night Eating Syndrome
**Sleep Quality**	Pearson correlation	0.638 **			
	Sig. (2-tailed)	0.0001			
**Circadian Patterns**	Pearson correlation	−0.712 **	−0.643 **		
	Sig. (2-tailed)	0.0001	0.0001		
**Night Eating Syndrome**	Pearson correlation	0.581 **	0.609 **	−0.702 **	
	Sig. (2-tailed)	0.0001	0.0001	0.0001	
**Age**	Pearson correlation	0.095	0.035	0.094	−0.059
	Sig. (2-tailed)	0.082	0.522	0.085	0.279
**BMI**	Pearson correlation	0.398 **	0.035	−0.456 **	0.483 **
	Sig. (2-tailed)	0.0001	0.522	0.0001	0.0001

** Correlation is significant at the 0.01 level (2-tailed).

**Table 4:** Best-fitting multiple linear regression model of older adults.

Items		Unstandardized Coefficients		Standardized Coefficients	*t*-Test	*p*-Value	Tolerance	VIF
		Beta Coefficient	Std. Error	β				
	**(Constant)**	83.944						
(a) For MES *	**Gender**	−1.025	1.127	−0.030	0.909	0.364	0.820	1.219
	**Residence**	6.929	1.225	0.207	5.655	0.000	0.688	1.454
	**Education**	−0.356	0.285	−0.044	0.251	0.212	0.757	1.321
	**Current job**	2.119	1.606	0.048	1.320	0.188	0.703	1.422
	**BMI**	−0.331	0.150	−0.081	0.200	0.028	0.679	1.473
	**Global PSQI score**	−0.698	0.204	−0.147	0.419	0.001	0.498	2.009
	**Night eating score**	−4.142	0.681	−0.270	6.084	0.0001	0.468	2.137
	**GSS**	−1.089	0.133	−0.346	0.198	0.0001	0.517	1.935
(b) For NES **	**(Constant)**	0.746						
	**Gender**	0.022	0.086	0.010	0.258	0.796	0.854	1.171
	**Residence**	−0.252	0.097	−0.115	0.589	0.010	0.656	1.525
	**Current job**	0.102	0.116	0.035	0.874	0.383	0.805	1.242
	**BMI**	0.048	0.011	0.181	4.270	0.000	0.724	1.381
	**MES**	−0.024	0.004	−0.372	0.981	0.000	0.336	2.980
	**Global PSQI score**	0.074	0.016	0.240	4.779	0.000	0.515	1.941
	**GSS**	0.012	0.011	0.061	1.106	0.270	0.430	2.324

* r-square = 0.69 Model ANOVA: F = 94.8, *p* = 0.0001; ** r-square = 0.57 Model ANOVA: F = 62.9, *p* = 0.0001.

**Table 5:** Bootstrap analysis of the mediating effect test.

Mediation Path					Estimate (B)	Std. Error	z-Value	*p*-Value	95% Confidence Interval	
									Lower	Upper
SV → SQ → CPs										
**Indirect**	GSS	→	PSQI	→ MES	−0.038	0.006	6.213	<0.001	−0.051	−0.026
**Direct**	GSS	→	MES		−0.096	0.009	10.902	<0.001	−0.116	−0.076
**Total**	GSS	→	MES		−0.134	0.007	18.569	<0.001	−0.147	−0.119
SV→ SQ → NES										
**Indirect**	GSS	→	PSQI	→ NEDS	0.053	0.008	6.75	<0.001	0.037	0.068
**Direct**	GSS	→	NEDS		0.066	0.011	6.082	<0.001	0.045	0.088
**Total**	GSS	→	NEDS		0.119	0.009	13.087	<0.001	0.101	0.137

**Table 6:** Standard path coefficients from the GSS predicting MES and NES through PSQI.

			Estimate (B)	Std. Error	z-Value	*p*-Value	95% Confidence Interval	
							Lower	Upper
***PSQI***	***→***	***MES***	−0.32	0.047	6.81	<0.001	−0.410	−0.222
**GSS**	**→**	**MES**	−0.1	0.009	10.9	<0.001	−0.116	−0.076
**GSS**	**→**	**PSQI**	0.120	0.008	15.202	<0.001	0.106	0.135
***PSQI***	***→***	***NES***	0.12	0.016	7.535	<0.001	0.092	0.157
**GSS**	**→**	**NES**	0.07	0.011	6.082	<0.001	0.045	0.088
**GSS**	**→**	**PSQI**	0.42	0.028	15.202	<0.001	0.369	0.478

## Data Availability

Data are available from the corresponding authors upon reasonable request.

## Abbreviations

BMI	Body Mass Index
GSS	Global Seasonality Scale
MEQ	Morningness–Eveningness Questionnaire
NEDQ	Night Eating Diagnostic Questionnaire
NES	Night Eating Syndrome
PHQ-9	Patient Health Questionnaire-9
PSQI	Pittsburgh Sleep Quality Index
SQ	Sleep Quality
SV	Seasonal Variation
CP	Circadian Pattern
